# Supplementary material for: Analysis of Codon Usage Patterns of Six Sequenced Brachypodium distachyon Lines Reveals a Declining CG Skew of the CDSs from the 5′-ends to the 3′-ends
Source: Genes (Basel). 2021 Sep 23;12(10):1467. doi: 10.3390/genes12101467 (PMC8535453; doi:10.3390/genes12101467)
Supplement: Supplementary file 1 [file genes-12-01467-s001.zip › genes-1307529 - supplementary.pdf]

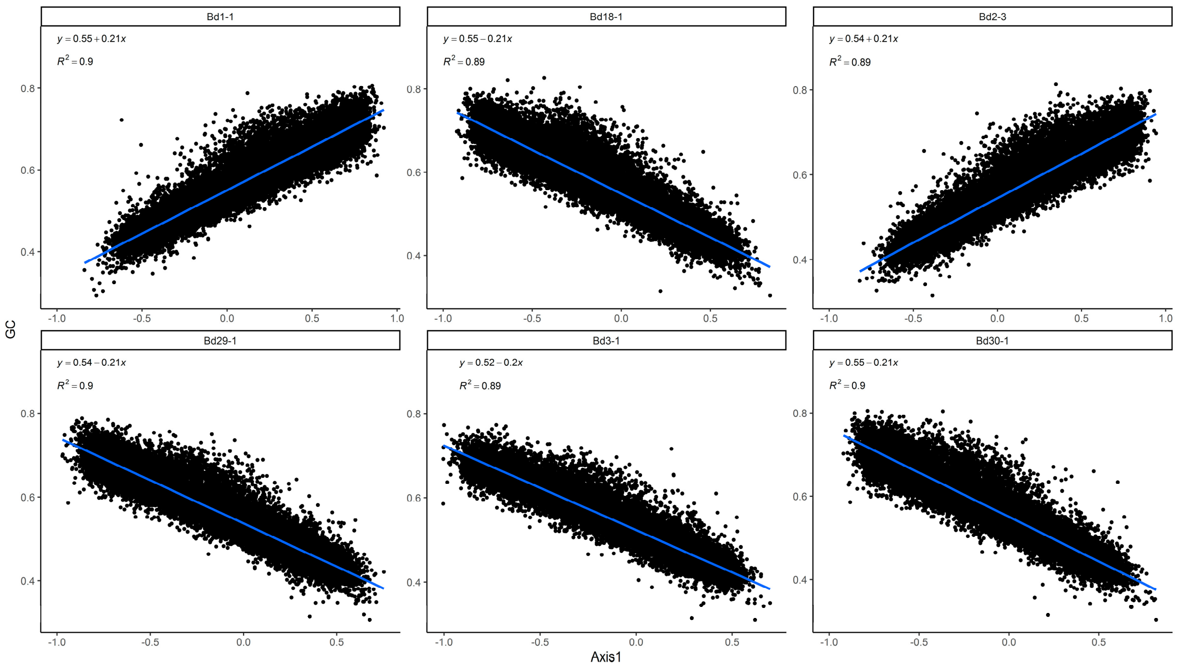

Figure S1: The correlations between the GC contents of genes in the six species and theirs positions on the first axis.

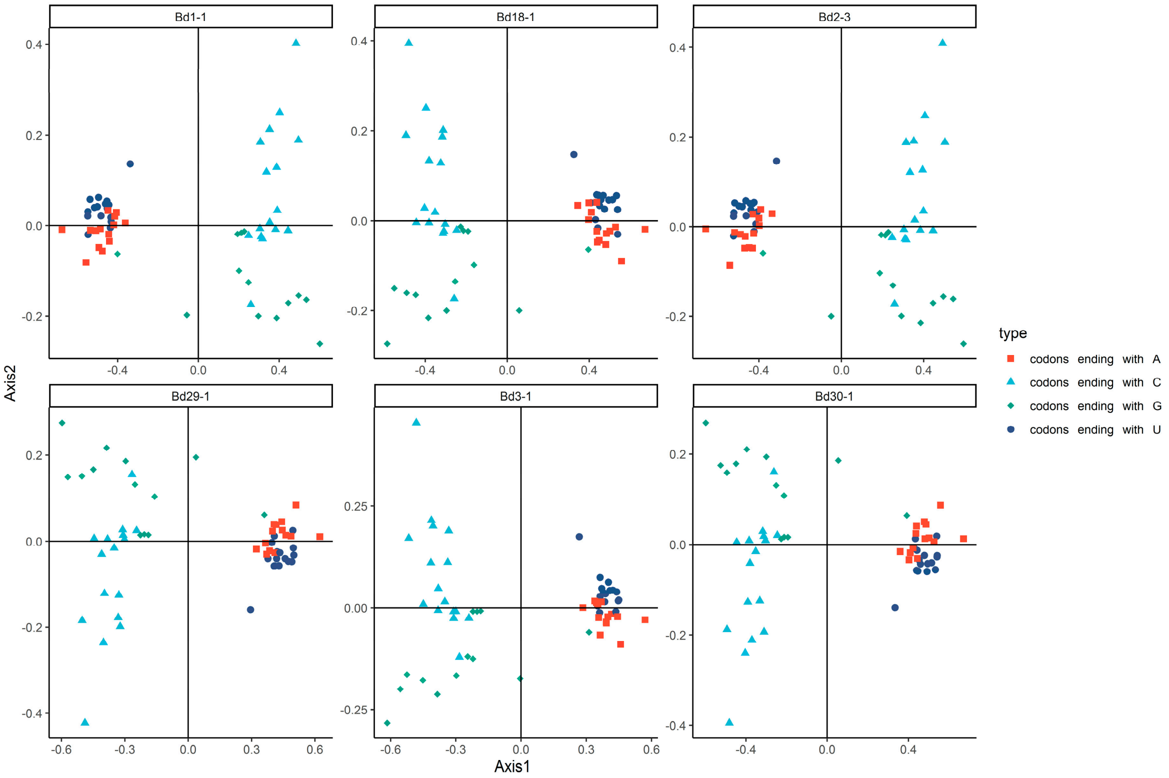

Figure S2. The distributions of codons from the six lines on the two axes.
